# Association of the Labor Migration of Parents With Nonsuicidal Self-injury and Suicidality Among Their Offspring in China

**DOI:** 10.1001/jamanetworkopen.2021.33596

**Published:** 2021-11-09

**Authors:** Ying Ma, Hongda Guo, Shuangshuang Guo, Ting Jiao, Chenyue Zhao, Brooke A. Ammerman, Martin M. Gazimbi, Yizhen Yu, Ruoling Chen, Harry H. X. Wang, Jie Tang

**Affiliations:** 1 Department of Child Healthcare, Guangzhou Women and Children’s Medical Center, Guangzhou Medical University, Guangzhou, China; 2 Department of Preventive Medicine, School of Public Health, Guangzhou Medical University, Xinzao, Panyu District, Guangzhou, China; 3 Department of Child and Adolescent Psychiatry, New York University School of Medicine, New York; 4 Department of Psychology, University of Notre Dame, Notre Dame, Indiana; 5 Department of Criminology and Sociology, University of Hull, Hull, United Kingdom; 6 Department of Maternal and Child Healthcare, School of Public Health, Tongji Medical College, Huazhong University of Science and Technology, Hankou District, Wuhan, China; 7 Faculty of Education, Health & Wellbeing, University of Wolverhampton, Wolverhampton, United Kingdom; 8 School of Public Health, Sun Yat-Sen University, Guangzhou, China

## Abstract

**Importance:**

The labor migration of parents in China often leaves children behind, which may be adversely associated with children’s health. However, few studies have assessed the association of parental migration with nonsuicidal self-injury (NSSI) or with suicidality among their offspring.

**Objective:**

To examine the associations of parental labor migration with NSSI and with suicidality as well as potential differential associations by sex among offspring left behind.

**Design, Setting, and Participants:**

This nationwide cross-sectional study was conducted from February to October 2015 among individuals aged 11 to 20 years from 45 public middle and high schools across 5 provinces of China. Data analysis was performed from November 1, 2020, to March 1, 2021.

**Exposures:**

Parental labor migration, including parental migration status (yes vs no), migration pattern (father, mother, or both), and the child’s age at the initial parent-child separation.

**Main Outcomes and Measures:**

Less frequent (1-4 episodes) NSSI, more frequent (≥5 episodes) NSSI, suicidal ideation, and suicide attempt in the past year were measured using validated questionnaires. Multinomial or binomial logistic regression analyses were used separately to estimate adjusted odds ratios (aORs) and 95% CIs of the associations between parental migration and NSSI, suicidal ideation, or suicide attempt. Potential covariates (demographic, family-level, and psychological characteristics) were adjusted for in 3 sequential models.

**Results:**

A total of 15 312 participants (7904 male [51.6%] and 7408 female [48.4%]) aged 11 to 20 years (mean [SD] age, 15.1 [1.8] years) were included. Of those participants, 5963 (23.3%) experienced parental migration. The 12-month prevalence of less frequent NSSI was 17.2% (2635 of 15 312), the 12-month prevalence of more frequent NSSI was 11.6% (1783 of 15 312), the 12-month prevalence of suicidal ideation was 15.2% (2335 of 15 312), and the 12-month prevalence of suicide attempt was 3.5% (535 of 15 312). Parental migration was associated with less frequent NSSI (adjusted odds ratio [aOR], 1.13; 95% CI, 1.03-1.24); no significant association of parental migration with more frequent NSSI (aOR, 1.01; 95% CI, 0.90-1.13), suicidal ideation (aOR, 1.00; 95% CI, 0.90-1.10), or suicide attempt (aOR, 1.09; 95% CI, 0.90-1.33) was identified. Compared with children whose parents did not migrate, the aOR for less frequent NSSI for participants whose father migrated was 1.18 (95% CI, 1.06-1.31), and the aOR for less frequent NSSI for participants having both parents migrate was 1.12 (95% CI, 1.01-1.28). Compared with children whose parents did not migrate, participants who experienced initial separation from 1 or both parents at preschool age had an aOR for less frequent NSSI of 1.16 (95% CI, 1.03-1.31). No sex disparities were found in these associations except for participants who experienced initial separation from 1 or both migrant parents at preschool age, for which the aOR for more frequent NSSI was higher among male (aOR, 1.27; 95% CI, 1.04-1.55) than female (aOR, 0.96; 95% CI, 0.77-1.19) participants.

**Conclusions and Relevance:**

This cross-sectional study found that parental migration, mainly of the father or of both parents, or an initial separation of children at preschool age from 1 or both parents who migrated was associated with higher odds of experiencing 1 to 4 NSSI episodes in 1 year among offspring. Overall, the associations of parental migration with NSSI and suicidality were similar between male and female participants.

## Key Points

**Question:**

Is the labor migration of parents in China associated with nonsuicidal self-injury (NSSI) or suicidality among children, adolescents, and young adults who were left behind, and are there sex differences in any association?

**Findings:**

In this cross-sectional study in China of 15 312 participants, labor migration of the father or of both parents was associated with 1 to 4 (but not ≥5) episodes of NSSI in 1 year and with suicidality among offspring, whereas differential associations by sex were minimal. In addition, higher odds of experiencing 1 to 4 episodes of NSSI were found for offspring who were initially separated from 1 or both parents at preschool age.

**Meaning:**

These findings suggest that interventions to address NSSI should consider potential associations with parental migration.

## Teaser

This cross-sectional study examines whether migration of 1 or both parents is associated with risk of nonsuicidal self-injury or suicidality and whether the risk differs by sex among the children, adolescents, and young adult offspring left behind in China.

## Introduction

Considered major public health concerns for adolescents worldwide, nonsuicidal self-injury (NSSI), suicidal ideation, and suicide attempt^1,2^ are not only associated with increased mental health risk (eg, depressive symptoms, hopelessness, and symptoms of borderline personality disorder)^3^ but are also the greatest risk factors associated with future suicidal behaviors.^4^ Thus, there is an urgent need to identify high-risk groups and specific risk factors for NSSI, suicidal ideation, and suicide attempt.

Labor migration refers to individuals who originate from low-waged areas and relocate in search of higher-waged employment opportunities, either internationally or domestically. As a result of labor migration, the migrants’ household income often increases and their families’ circumstances improve. However, their children are often left behind in the care of other family members or caregivers owing to the transient nature of the work or to financial constraints.^5^ Given these circumstances, numerous studies have examined the association of parental migration with the well-being of children.^6,7^ A recent meta-analysis suggested that, compared with children of parents who did not migrate, children left behind by parents who migrated for employment had increased risk of depression, anxiety, suicidal ideation, conduct disorder, substance use, wasting, and stunting.^7^ Despite these findings, few studies have specifically focused on the the health outcomes of NSSI,^8,9,10,11^ suicidal ideation, or suicide attempt.^12,13,14,15^ Moreover, findings that do exist are mixed and inconclusive. Although some studies have found that parental migration was associated with increased risk of NSSI^9,11^ and suicidal ideation^12,13,14^ among children, other studies indicated null or even inverse associations of parental migration with risk of NSSI,^8,10^ suicidal ideation,^15^ and suicide attempt.^12,15^ In addition, most studies comparing the risk of NSSI between children who were and children who were not left behind did not adjust for potential confounders,^9,10,11,13^ such as socioeconomic status and emotional regulation factors (eg, loneliness, emotional management ability, and psychological resilience)^16^; therefore, the independent association between parental migration and NSSI remains uncertain.

One previous study reported that a lack of parental emotional support—a consequence for many children who are left behind—had a stronger negative association with hippocampal growth at preschool age than at school age and early adolescence,^17^ suggesting that there may be a time window for the outcomes associated with parent-child separation due to labor migration. However, to our knowledge, no studies have explored whether the associations of parental migration with NSSI, suicidal ideation, or suicide attempt may vary with the child’s age at the initial parent-child separation. It is of substantial public health importance to address this research gap to inform strategies for NSSI and suicidality prevention as well as to provide evidence for optimizing policies to protect labor migrants’ rights.

The present study investigated the possible associations of parental labor migration (yes or no), which parent migrated (father, mother, or both), and the age of the child at the initial parent-child separation (preschool age, school age, or adolescence) with NSSI, suicidal ideation, and suicide attempt among offspring. Because previous studies have shown sex differences in NSSI and suicidality prevalence^1^ and in the association between parental migration and health behaviors in children,^12^ we also aimed to understand whether there were sex differences in the associations of parental migration with NSSI and suicidality.

## Methods

### Study Participants

We used data from a nationwide survey among middle school and high school students in rural China that aimed to explore the epidemiologic characteristics and risk factors of adolescent behavioral problems and provide evidence for policymaking. The survey’s design, procedure, and implementation have been described previously.^18,19^ In brief, using a stratified cluster random sampling method, a representative sample of 15 797 students from 27 middle schools and 18 high schools in 5 provinces (Heilongjiang, Hubei, Anhui, Guangdong, and Yunnan provinces) across different regions of China were selected to participate in the survey from February to October 2015. The present study excluded participants who were orphans,^20^ did not answer the items regarding parental migration, or did not complete assessments of NSSI, suicidal ideation, or suicide attempt. This study followed the American Association for Public Opinion Research (AAPOR) reporting guideline and received ethics clearance from Guangzhou Medical University. Before participation in the survey,^19^ all students or their guardians (if students were younger than 14 years) provided written informed consent that was obtained in a manner consistent with the Declaration of Helsinki.^21^ No one received compensation or was offered any incentive for participating in this study.

### Instruments

#### Measurement of Sociodemographic Profile, Parental Migration, Loneliness, Psychological Resilience, Emotional Management Ability, and Social Support

We used a custom-designed questionnaire to collect demographic, family, and parenting characteristics, including age, sex, ethnicity (Han or others, meaning, every category of ethnicity except Han), single-child family (yes or no), single-parent family (yes or no), educational level of the main caregiver (middle school or below, high school or technical school, college or above), family income (<$150/month, $150-850/month, or >$850/month), and parenting style (strict, pampered, neglect or frequently changing, or open-minded). The test-retest reliability of the questionnaire was α = .83.^22^

Parental migration was considered “yes” in response to the question “Have/did your father or/and mother migrated to an urban area for employment and not living with you for at least half a year?”^23^ For respondents who answered yes to parental migration, the survey asked which parent migrated (father, mother, or both) and the child’s age at the initial separation. The parental migration pattern was categorized into 3 groups (mother, father, or both parents) in response to the question “Which of your parent(s) migrated to urban area for employment and were not living with you for at least half a year?” The age of the child at the initial separation from parents who migrated was categorized as preschool age (<6 years old), school age (6-10 years old), or adolescence (>10 years old) in response to the question “How old were you when 1 or both of your parents migrated to urban area for employment (the earliest time you know)?” The Cronbach α coefficient for these questions in the present study was 0.73.

The revised version of the Loneliness Scale was used to measure loneliness.^24^ It consists of 21 items with 5-point Likert-type responses (1 = fully inconsistent; 2 = inconsistent; 3 = not sure; 4 = consistent; and 5 = fully consistent). A higher total score indicates greater loneliness.^24^ The Cronbach α coefficient for the scale in the present study was 0.78.

We measured psychological resilience using the Resilience Scale for Chinese Adolescents, which has 27 items with 5-point Likert-type responses (1 = fully inconsistent; 2 = inconsistent; 3 = not sure; 4 = consistent; and 5 = fully consistent).^25^ A higher total score indicates better psychological resilience. The Cronbach α coefficient of the scale in the present study population was 0.76.

To measure emotional management ability, we used a 4-item subscale of the Emotional Intelligence inventory,^26^ which has 4-point Likert-type responses (1 = always like this; 2 = often like this; 3 = rarely like this; and 4 = never like this). Higher total scores represent greater emotional management ability. The Cronbach α coefficient of this subscale in the present study was 0.78.

Social support of the participants was measured using the 17-item Adolescent Social Support Scale (eTable 1 in the Supplement),^27^ which has 5-point Likert-type responses for each item (1 = strongly agree; 2 = agree; 3 = neutral; 4 = somewhat disagree; 5 = strongly disagree). The Cronbach α coefficient of the scale in the present study was 0.93.

#### Measurements of NSSI, Suicidal Ideation, and Suicide Attempt

The Chinese version of the Functional Assessment of Self-mutilation was used to assess for method, frequency, and purpose of NSSI during the past 12 months.^28^ Participants were asked, “During the past 12 months, have you harmed yourself in a way that was deliberate, but not intended to take your life?” A list of 8 NSSI methods were specified, including hitting, head banging, stabbing, pinching, scratching, biting, burning, and cutting. For these participants who confirmed that they had engaged in NSSI, the frequency of NSSI was asked. Similar to previous studies,^19,28,29^ participants were divided into 3 categories based on the total frequency across the 8 forms of NSSI in the past 12 months: (1) engaged in NSSI 5 or more times, defined as more frequent NSSI^30^; engaged in NSSI 1 to 4 times, defined as less frequent NSSI,^27,28^ and (3) did not engaged in NSSI, termed non-NSSI. The internal consistency reliability of this assessment in the present study was α = .82.

Suicidal ideation and suicide attempt were measured using items derived from the Global School-Based Student Health Survey.^31^ Suicidal ideation was defined as an affirmative answer to the question “During the past 12 months, did you ever seriously consider attempting suicide?” Suicide attempt was measured by the question “During the past 12 months, how many times did you actually attempt suicide?” The responses were categorized as 0 times vs 1 or more times.

### Statistical Analysis

The data analysis was performed from November 1, 2020, to March 1, 2021. Frequencies and proportions for categorical variables or mean (SD) values for continuous variables were used to describe participant characteristics and NSSI or suicidality by study variables. We used the χ^2^ test or a 2-tailed unpaired *t* test to compare the distribution between participants of parental migration and nonmigration according to study variables.

To examine the associations of parental migration with NSSI and suicidality, we first used multinomial or binomial logistic regression analyses to separately estimate the adjusted odds ratios (aORs) and 95% CIs of parental migration for participants who engaged in NSSI (less frequent NSSI and more frequent NSSI), suicidal ideation, and suicide attempt. Different covariates were adjusted for in 3 models. In model 1, we adjusted for the demographic characteristics of participants, including province, age (continuous data), sex, and ethnicity. In model 2, we additionally adjusted for covariates of family-level characteristics, including main caregiver’s educational level, single-child family, single-parent family, family income, parenting styles, and social support (continuous data). In model 3, we additionally adjusted for psychological covariates, including loneliness (continuous data), psychological resilience (continuous data), and emotional management ability (continuous data). In secondary analyses, the same regression models were used to separately examine the associations of parental migration pattern (ie, which parent migrated) and child age at initial child-parent separation with NSSI, suicidal ideation, and suicide attempt.

We conducted subgroup analyses to examine whether sex differences in the associations of parental migration with NSSI and suicidality emerged. Differences were assessed by calculating a ratio of the ORs.^32^

We imputed missing data of continuous covariables based on mean values, and we imputed missing data of categorical covariables using a separate category.^33^ The statistical significance level for the primary analyses was *P* < .05. To reduce the potential for type I errors due to multiple comparisons in secondary and subgroup analyses, we adjusted the statistical significance level using the Bonferroni method.^34^ All tests were 2-sided, and all analyses were conducted using IBM SPSS Statistics, version 25.0 (IBM Corp).

## Results

Of 15 797 students, 78 did not provide the consent form, 21 were absent from school on the day of the survey, and 75 submitted an incomplete questionnaire with at least 15% of the items unanswered. The final sample included 15 623 students, a response rate of 98.9%. We then excluded 33 participants who were orphans and 278 participants who did not respond to any item regarding parental migration. The remaining 15 312 participants (7904 male [51.6%] and 7408 female [48.4%]) were included in the present analysis, with approximately equal distribution across the 5 study provinces. The age of the participants ranged from 11 to 20 years (mean [SD] age, 15.1 [1.8] years). More than half (8161 [53.3%]) of the participants were middle school students, 13 860 (90.5%) were Han ethnicity, 5290 (34.5%) were a single child, and 754 (4.9%) were from single-parent families. In total, 5963 participants (38.9%) experienced parental migration, 3575 (23.3%) experienced migration of the father, 480 (3.1%) experienced migration of the mother, and 1908 (12.5%) experienced migration of both parents. The percentages of participants who were initially separated from their parents were 17.4% (2665 of 15 312) at preschool age, 15.2% (2330 of 15 312) at school age, and 6.3% (968 of 15 312) at adolescence. Additional characteristics are presented in Table 1.

**Table 1.** Characteristics of Participants by the Presence or Absence of Parental Migration

Characteristic^a^	Participants, No. (%)						
	Parental migration		Total (N = 15 312)	Nonsuicidal self-injury		Suicidal ideation (n = 2335)	Suicide attempt (n = 535)
	No (n = 9349)	Yes (n = 5963)		Less frequent (n = 2635)^b^	More frequent (n = 1783)^c^		
Age, mean (SD), y	15.1 (1.8)	15.2 (1.9)	15.1 (1.8)	15.1 (1.9)	15.0 (1.8)	14.9 (1.7)	14.8 (1.6)
Sex							
Male	4795 (51.3)	3109 (52.1)	7904 (51.6)	1359 (51.6)	881 (49.4)	1288 (52.6)	277 (51.8)
Female	4554 (48.7)	2854 (47.9)	7408 (48.4)	1276 (48.4)	902 (50.6)	1107 (47.4)	258 (48.2)
Province							
Heilongjiang	2046 (21.9)	7.5 (11.8)	2751 (18.0)	342 (13.0)	161 (9.0)	304 (13.0)	61 (11.4)
Anhui	1459 (15.6)	1843 (30.9)	3302 (21.6)	655 (24.9)	395 (22.2)	496 (21.2)	116 (21.7)
Hubei	1291 (13.8)	1650 (27.7)	2941 (19.2)	546 (20.7)	341 (19.1)	487 (20.9)	112 (20.9)
Guangdong	1880 (20.1)	1107 (18.6)	2978 (19.5)	592 (22.5)	368 (20.6)	480 (20.6)	88 (16.4)
Yunnan	2673 (28.6)	658 (11.0)	3331 (21.8)	500 (19.0)	518 (29.1)	568 (24.3)	158 (29.5)
Educational level							
Junior high school	5216 (55.8)	2945 (49.4)	8161 (53.3)	1388 (52.7)	999 (56.0)	1349 (57.8)	345 (64.5)
Senior high school	4133 (44.2)	3018 (50.6)	7151 (46.7)	1247 (47.3)	784 (44.0)	986 (42.2)	190 (35.5)
Ethnicity							
Han	8171 (87.4)	5689 (95.4)	13860 (90.5)	2428 (92.1)	1524 (85.5)	2066 (88.5)	447 (83.6)
Other^d^	1178 (12.6)	274 (4.6)	1452 (9.5)	207 (7.9)	259 (14.5)	269 (11.5)	88 (16.4)
Educational level of main caregiver							
College or above	884 (9.5)	168 (2.8)	1052 (6.9)	158 (6.0)	147 (8.2)	192 (8.2)	63 (11.8)
Senior middle school or technical school	2266 (24.2)	1013 (17.0)	3279 (21.4)	576 (21.9)	409 (22.9)	515 (22.1)	104 (19.4)
Junior middle school or below	6033 (64.5)	4717 (79.1)	10 750 (70.2)	1873 (71.1)	1205 (67.6)	1599 (68.5)	363 (67.9)
Single-child family	3707 (39.7)	1583 (26.5)	5290 (34.5)	843 (32.0)	563 (31.6)	813 (34.8)	179 (33.5)
Single-parent family	407 (4.4)	347 (5.8)	754 (4.9)	117 (4.4)	114 (6.4)	141 (6.0)	29 (5.4)
Family income, $/mo							
<150	1620 (17.3)	1029 (17.3)	2649 (17.3)	448 (17.0)	338 (19.0)	413 (17.7)	108 (20.2)
150-850	6465 (69.2)	4163 (69.8)	10628 (69.4)	1854 (70.4)	1193 (66.9)	1563 (66.9)	332 (62.1)
>850	1264 (13.5)	771 (12.9)	2035 (13.3)	333 (12.6)	252 (14.1)	359 (15.4)	95 (17.8)
Parenting style^e^							
Strict	2873 (30.7)	1769 (29.7)	4642 (30.3)	766 (29.1)	545 (30.6)	655 (28.1)	163 (30.5)
Pamper	295 (3.2)	253 (4.2)	548 (3.6)	103 (3.9)	73 (4.1)	91 (3.9)	15 (2.8)
Neglect or frequently changing	937 (10.0)	710 (11.9)	1647 (10.8)	353 (13.4)	280 (15.7)	432 (18.5)	122 (22.8)
Open-minded	4861 (52.0)	2996 (50.2)	7857 (51.3)	1316 (49.9)	824 (46.2)	1059 (45.5)	212 (39.6)
Missing data	383 (4.1)	235 (3.9)	618 (4.0)	97 (3.7)	61 (3.4)	98 (4.2)	23 (4.3)
Loneliness score, mean (SD)	49.3 (10.1)	50.8 (10.4)	49.9 (10.2)	51.6 (10.1)	54.6 (11.0)	55.2 (11.4)	56.5 (12.9)
Psychological resilience score, mean (SD)	92.7 (13.1)	91.1 (12.9)	92.1 (13.1)	90.3 (12.3)	87.7 (12.4)	86.0 (12.3)	84.5 (12.3)
Emotional management ability score, mean (SD)	11.6 (2.8)	11.4 (2.9)	11.5 (2.8)	10.9 (2.6)	10.2 (3.0)	10.1 (3.0)	9.9 (3.3)
Social support score, mean (SD)	63.8 (14.2)	62.1 (14.3)	63.1 (14.3)	60.9 (13.9)	57.5 (14.6)	56.3 (14.9)	55.3 (16.1)

^a^ The distributions of parental migration with respect to this characteristic were all statistically significant (*P* < .05), except for family income.

^b^ Includes 1 to 4 episodes.

^c^ Includes 5 or more episodes.

^d^ Includes all ethnicities except Han.

^e^ Missing data.

Of 15 312 participants, the 12-month prevalence of less frequent NSSI was 17.2% (2635 participants); more frequent NSSI, 11.6% (1783 participants); suicidal ideation, 15.3% (2335 participants); and suicide attempt, 3.5% (535 participants) (Table 1). Of the 28.8% (4418 of 15 312) of the overall sample of respondents who engaged in NSSI at least once in the 12 months preceding the survey, 94.1% (4159 of 4418) of the respondents with self-injury reported engaging in 1 to 5 types of NSSI (mean [SD] number, 2.4 [1.6]; median, 2.0; range, 1-8 types). Self-hitting, self-pinching, and self-stabbing were the most frequent types of NSSI reported in the study population (eTable 2 in the Supplement).

Participants who experienced parental migration reported an increased risk of less frequent and of more frequent NSSI, suicidal ideation, and suicide attempt (Table 2). Significant associations were found between parental migration status (ie, whether or not a parent migrated) and less frequent NSSI in all models. In the fully adjusted model (model 3) for less frequent NSSI, the aOR for parental migration was 1.13 (95% CI, 1.03-1.24). No significant associations of parental migration with more frequent NSSI, suicidal ideation, or suicide attempt were found in any model (Table 2). Subgroup analysis indicated no sex differences in the associations of parental migration with NSSI or suicidality (eTable 3 in the Supplement).

**Table 2.** Odds of NSSI, Suicidal Ideation, and Suicide Attempt by Parental Migration Status

Variable	Participants, No. (%)	Odds ratio (95% CI)			
		Unadjusted	Model 1^a^	Model 2^b^	Model 3^c^
Less frequent (1-4 episodes) NSSI					
No migration	1483 (15.9)	1 [Reference]	1 [Reference]	1 [Reference]	1 [Reference]
Migration	1152 (19.3)	1.28 (1.18-1.40)	1.18 (1.08-1.30)	1.15 (1.04-1.26)	1.13 (1.03-1.24)
More frequent (≥5 episodes) NSSI					
No migration	1081 (11.6)	1 [Reference]	1 [Reference]	1 [Reference]	1 [Reference]
Migration	702 (11.8)	1.08 (0.97-1.19)	1.10 (0.98-1.22)	1.04 (0.93-1.17)	1.01 (0.90-1.13)
Suicidal ideation					
No migration	1410 (15.1)	1 [Reference]	1 [Reference]	1 [Reference]	1 [Reference]
Migration	925 (15.5)	1.03 (0.95-1.13)	1.03 (0.94-1.14)	1.02 (0.92-1.12)	1.00 (0.90-1.10)
Suicide attempt					
No migration	316 (3.4)	1 [Reference]	1 [Reference]	1 [Reference]	1 [Reference]
Migration	219 (3.7)	1.09 (0.91-1.30)	1.15 (0.96-1.39)	1.11 (0.92-1.35)	1.09 (0.90-1.33)

Abbreviation: NSSI, nonsuicidal self-injury.

^a^ Adjusted for province, age, ethnicity, and sex.

^b^ Additionally adjusted for single-child family, single-parent family, educational level of main caregiver, family income, parenting style, and social support.

^c^ Additionally adjusted for offspring loneliness, psychological resilience, and emotional management ability scores.

Compared with participants of parents who did not migrate, the unadjusted ORs and aORs for less frequent NSSI were significantly increased for participants with migration of the father or both parents but not with migration of the mother (Table 3). In the fully adjusted model for less frequent NSSI (model 3), the aOR was 1.18 (95% CI, 1.06-1.31) for migration of the father, 1.12 (95% CI, 1.01-1.28) for migration of both parents, and 0.84 (95% CI, 0.64-1.10) for migration of the mother. There was no significant association between which parent migrated and more frequent NSSI, suicidal ideation, or suicide attempt. Similarly, no sex differences were found in the associations between which parent migrated and NSSI or suicidality (eTable 4 in the Supplement).

**Table 3.** Odds of NSSI, Suicidal Ideation, and Suicide Attempt by Parental Migration Type

Variable	Participants, No. (%)	Odds ratio (95% CI)			
		Unadjusted	Model 1^a^	Model 2^b^	Model 3^c^
Less frequent NSSI					
Migration					
None	1483 (15.9)	1 [Reference]	1 [Reference]	1 [Reference]	1 [Reference]
Father	711 (19.9)	1.33 (1.20-1.47)	1.22 (1.10-1.36)	1.20 (1.08-1.34)	1.18 (1.06-1.31)
Mother	71 (14.8)	0.93 (0.72-1.21)	0.86 (0.66-1.12)	0.84 (0.64-1.09)	0.84 (0.64-1.10)
Both parents	370 (19.4)	1.29 (1.14-1.47)	1.20 (1.05-1.37)	1.13 (1.01-1.28)	1.12 (1.01-1.28)
More frequent NSSI					
Migration					
None	1081 (11.6)	1 [Reference]	1 [Reference]	1 [Reference]	1 [Reference]
Father	416 (11.6)	1.07 (0.94-1.21)	1.08 (0.95-1.23)	1.05 (0.92-1.20)	1.01 (0.88-1.15)
Mother	59 (12.3)	1.06 (0.80-1.40)	1.06 (0.79-1.41)	1.01 (0.76-1.36)	1.00 (0.74-1.34)
Both parents	227 (11.9)	1.08 (0.93-1.27)	1.13 (0.96-1.32)	1.04 (0.93-1.18)	1.02 (0.85-1.19)
Suicidal ideation					
Migration					
None	1410 (15.1)	1 [Reference]	1 [Reference]	1 [Reference]	1 [Reference]
Father	555 (15.5)	1.04 (0.93-1.15)	1.03 (0.92-1.15)	1.01 (0.90-1.14)	0.97 (0.86-1.09)
Mother	64 (13.3)	0.87 (0.66-1.34)	0.87 (0.66-1.14)	0.84 (0.63-1.11)	0.82 (0.61-1.09)
Both parents	306 (16.0)	1.08 (0.94-1.23)	1.10 (0.96-1.26)	1.02 (0.88-1.17)	1.00 (0.84-1.13)
Suicide attempt					
Migration					
None	316 (3.4)	1 [Reference]	1 [Reference]	1 [Reference]	1 [Reference]
Father	126 (3.5)	1.04 (0.85-1.29)	1.07 (0.86-1.34)	1.05 (0.84-1.32)	1.00 (0.79-1.25)
Mother	22 (4.6)	1.37 (0.88-2.14)	1.46 (0.93-2.27)	1.52 (0.97-2.38)	1.53 (0.97-2.42)
Both parents	71 (3.7)	1.11 (0.85-1.44)	1.23 (0.94-1.61)	1.13 (0.86-1.49)	1.09 (0.82-1.44)

Abbreviation: NSSI, nonsuicidal self-injury.

^a^ Adjusted for participant province, age, ethnicity, and sex.

^b^ Additionally adjusted for single-child family, single-parent family, educational level of main caregiver, family income, parenting style, and social support.

^c^ Additionally adjusted for offspring loneliness, psychological resilience, and emotional management ability scores.

Participants who experienced initial separation from 1 or both parents before 6 years of age or between 6 and 10 years of age had a higher likelihood of less frequent NSSI than participants whose parents did not migrate (Table 4). However, in the fully adjusted model (model 3), a significant association was found only among participants who experienced initial separation from migrating parents before the age of 6 years (aOR, 1.16; 95% CI, 1.03-1.13). The unadjusted ORs and aORs in model 1 and model 2 for more frequent NSSI, suicidal ideation, and suicide attempt were also significantly higher among participants who experienced initial separation before 6 years of age from 1 or both parents who migrated. However, none of these ORs remained significant in the fully adjusted model (model 3). Subgroup analysis assessing sex showed that an initial separation before 6 years of age from 1 or both migrating parents was associated with higher odds of having more frequent NSSI among male participants (OR, 1.27; 95% CI, 1.04-1.55) than female participants (OR, 0.96; 95% CI, 0.77-1.19) (eTable 5 in the Supplement).

**Table 4.** Odds of Nonsuicidal Self-injury, Suicidal Ideation, and Suicide Attempt by Age of Offspring When Parent Initially Migrated

Variable	No. (%)	Odds ratio (95% CI)			
		Unadjusted	Model 1^a^	Model 2^b^	Model 3^c^
Less frequent NSSI					
No migration	1483 (15.9)	1 [Reference]	1 [Reference]	1 [Reference]	1 [Reference]
Preschool age (≤6 y)	539 (20.2)	1.39 (1.25-1.56)	1.24 (1.11-1.40)	1.19 (1.06-1.34)	1.16 (1.03-1.31)
School age (6-10 y)	440 (18.9)	1.22 (1.08-1.38)	1.15 (1.01-1.30)	1.11 (0.98-1.26)	1.10 (0.97-1.25)
Adolescence (>10 y)	173 (17.9)	1.15 (0.96-1.37)	1.12 (0.94-1.34)	1.10 (0.92-1.32)	1.11 (0.93-1.33)
More frequent NSSI					
No migration	1081 (11.6)	1 [Reference]	1 [Reference]	1 [Reference]	1 [Reference]
Preschool age (≤ 6 y)	357 (13.4)	1.27 (1.11-1.44)	1.26 (1.10-1.45)	1.17 (1.02-1.35)	1.11 (0.96-1.29)
School age (6-10 y)	240 (10.3)	0.91 (0.79-1.06)	0.96 (0.82-1.12)	0.91 (0.76-1.06)	0.89 (0.76-1.05)
Adolescence (>10 y)	105 (10.8)	0.96 (0.77-1.18)	1.00 (0.80-1.25)	1.00 (0.81-1.25)	1.01 (0.81-1.26)
Suicidal ideation					
No migration	1410 (15.1)	1 [Reference]	1 [Reference]	1 [Reference]	1 [Reference]
Preschool age (≤6 y)	478 (17.9)	1.23 (1.10-1.38)	1.21 (1.07-1.36)	1.14 (1.01-1.29)	1.07 (0.94-1.22)
School age (6-10 y)	329 (14.1)	0.93 (0.81-1.05)	0.94 (0.82-1.07)	0.91 (0.79-1.05)	0.90 (0.78-1.03)
Adolescence (>10 y)	118 (12.2)	0.78 (0.64-0.96)	0.82 (0.67-1.01)	0.83 (0.67-1.02)	0.83 (0.67-1.02)
Suicide attempt					
No migration	316 (3.4)	1 [Reference]	1 [Reference]	1 [Reference]	1 [Reference]
Preschool age (≤6 y)	115 (4.3)	1.29 (1.04-1.60)	1.35 (1.07-1.70)	1.27 (1.01-1.58)	1.15 (0.90-1.46)
School age (6-10 y)	67 (2.9)	0.85 (0.65-1.11)	0.90 (0.69-1.19)	0.88 (0.67-1.17)	0.87 (0.66-1.16)
Adolescence (>10 y)	37 (3.8)	1.14 (0.80-1.61)	1.26 (0.89-1.79)	1.28 (0.90-1.83)	1.32 (0.92-1.89)

^a^ Adjusted for province, age, ethnicity, and sex.

^b^ Additionally adjusted for single-child family, single-parent family, educational level of main caregiver, family income, parenting styles, and social support.

^c^ Additionally adjusted for offspring loneliness, psychological resilience, and emotional management ability scores.

## Discussion

Using data from a nationwide, school-based survey, this cross-sectional study investigated the associations of parental migration status, parental migration pattern (which parent migrated), and the age of the offspring at the initial parent-child separation with NSSI, suicidal ideation, and suicide attempt among Chinese children, adolescents, and young adults; differential associations between male and female participants were also assessed. Our findings suggested that parental migration was positively associated with less frequent NSSI but not more frequent NSSI, suicidal ideation, and suicide attempt in offspring. The data also indicated that migration of the father or of both parents, rather than migration of the mother, was associated with less frequent NSSI. Children initially separated at preschool age from 1 or both migrating parents were more likely to engage in less frequent NSSI than those who experienced the initial separation later in life. No significant sex differences were found in the associations of parental migration status, pattern, and child age at initial separation with NSSI and suicidality, except that early parent-child separation (when children were younger than 6 years) had increased risk of more frequent NSSI in male participants.

Four existing studies^8,9,10,11^ have examined the association of parental migration with NSSI in children and adolescents with mixed conclusions or less convincing findings due to the use of an ambiguous definition of NSSI^8^ or the failure to adjust for potential confounders.^9,10,11^ In the present study, we used a validated diagnostic criterion for NSSI^19,28,29^ and adjusted for up to 13 potential confounders, providing robust evidence that parental migration is independently associated with increased risk of less frequent NSSI among Chinese children, adolescents, and young adults and that those offspring who were left behind are at high risk for less frequent NSSI engagement.

Despite the variability in previous findings,^13,14^ our results were consistent with those of a previous study suggesting that the risks of suicidal ideation and suicide attempt were not significantly different between children who were and children who were not left behind.^15^ The discrepancies in the findings between studies for the associations of parental migration with NSSI and with suicidality may be related to the study location, apart from study design, study sample, and measurements. In China, more than one-third of children residing in rural areas (61 million) have at least 1 parent who migrated,^35^ making the health and well-being of these children a priority concern. The Chinese government has called on local authorities to take responsibility for the care of these children.^36^ However, the health care policies and actions for children who are left behind are different across provinces,^37^ making it difficult to ascertain their associations with the health and well-being of these children. The risks of NSSI and suicidality among children and adolescents who are left behind may be mitigated in places with well-formulated health care policies and actions.^7^ In light of China’s ongoing actions for children who are left behind, it may be unsurprising to see the nonsignificant association of parental migration with more frequent NSSI, suicidal ideation, and suicide attempt.

To our knowledge, only 1 existing study investigated sex differences in the association of parental migration with NSSI. Knipe and colleagues^8^ conducted a hospital-based case-control study and found no sex differences in self-poisoning behavior (the most common method of NSSI in Sri Lanka) between boys and girls with at least 1 parent who migrated, consistent with the findings of our study. Nonsuicidal self-injury for emotional regulation is closely associated with coping strategies,^38^ and no sex differences have been detected for coping strategies.^39^ Further investigations are warranted to better understand NSSI-specific coping mechanisms.

The association of parental migration with less frequent NSSI may be explained as follows. First, the caretakers of children and adolescents who are left behind have different family roles, educational levels, and lifestyles from the children’s own parents, which may be risk factors associated with an unfavorable environment for psychological development related to NSSI, such as depression, loneliness, and poor emotional management ability.^7,40^ Experimental and animal studies suggest that parent-child separation may disrupt gene expression in a critical stage of development,^41,42^ which may be associated with NSSI. Second, compared with migration of the mother, migration of the father appears to have greater association with less frequent NSSI, possibly because of different caregiving roles for fathers and mothers.^43^ Previous research showed that family poverty had a stronger negative association with the father’s emotional warmth and support than the mother’s,^44^ and migrating fathers were also associated with providing less warmth and emotional support than migrating mothers.^45^ Third, separation at preschool age from 1 or both migrating parents was more strongly associated with less frequent NSSI than separation at later years in a child’s life. This finding may be because early childhood is a sensitive period in human development during which the brain, especially the circuitry governing emotion, attention, self-control, and stress, is shaped by the interplay of the child’s biological and environmental factors.^17,46^ Further studies are needed to delineate specific developmental mechanisms and their associations with parental migration on NSSI.

### Strengths and Limitations

One major strength of our study is the sample representativeness. We recruited a large sample size of children, adolescents, and young adults across 5 provinces, with the social, economic, and cultures reflecting the status in rural China. In addition, the adjustment for a variety of potential confounders in multivariate analysis, as well as in subsequent subgroup analyses, reinforced the validity and robustness of our findings. Our results assessing the associations between NSSI, suicidal ideation, and suicide attempt and which parent migrated and the timing of the parent-child separation may inform the development of preventive interventions to address these concerns among children, adolescents, and young adults, particularly for NSSI, and the policies to support migrating families.

Our study has limitations. First, parental migration may be misclassified, and the prevalence of NSSI and suicidality may be underestimated or overestimated owing to recall bias. Nonetheless, the proportion of offspring who were left behind in our study was comparable to that reported to the National Bureau of Statistics of China.^47^ In addition, previous studies have shown that school-based self-reported data collection regarding self-harm, suicidality, and risk factors is likely to be reliable, and such data are valuable when prospective data are not available.^48^ Second, participants may not have the same capacity to understand the questionnaire due to a mixed sample that included students of all school grades, although study investigators were trained to use a standardized procedure. Third, we may have included several offspring from the same family in the survey, which would lead to underestimating or overestimating the association of parental migration with NSSI and suicidality, although the likelihood is low. Fourth, we did not assess depression, a potential confounder in the present study; however, we adjusted for loneliness, which is considered the strongest factor associated with depression.^49^ Moreover, although we extensively adjusted for potential confounders, we cannot rule out the possibility of residual confounders by unknown factors and other factors that were not assessed in this survey. Fifth, although we adjusted for social support and emotional management, we did not investigate the mediating and moderating mechanisms underlying the association of parental migration with NSSI and suicidality, which will be the direction of our future studies.

## Conclusions

The findings of our cross-sectional study suggesting that parental migration is independently associated with NSSI among offspring may have important public health implications. Thus, it is necessary to improve monitoring of NSSI and its known risk factors and implement early interventions with tailored support strategies for children, adolescents, and young adults who were left behind by migrating parents. Individuals with a father or both parents who migrated and children who were separated at preschool ages from 1 or both migrant parents may be particularly vulnerable. Findings from this study may also inform clinicians, teachers, and other stakeholders about potential health needs of this population and may contribute to specific policy initiatives for improving the behavioral health of offspring of migrating parents. Further investigation is warranted to develop a multidimensional intervention framework for NSSI and suicidality among children, adolescents, and young adults in the context of prolonged parent-child separation in families with parent migration.

## Notes

Supplement. **eTable 1.** Adolescent Social Support Scale **eTable 2.** Frequency and Prevalence of 8 Types of NSSI **eTable 3.** Odds of NSSI, Suicidal Ideation and Suicide Attempt by Migration Status in Male vs Female Participants **eTable 4.** Odds of NSSI, Suicidal Ideation and Suicide Attempt by Parental Migration Pattern in Male vs Female Participants **eTable 5.** Odds of NSSI, Suicidal Ideation and Suicide Attempt by Offspring Age When Parent Initially Migrated in Male vs Female Participants
