# Supplementary material for: Association of the Labor Migration of Parents With Nonsuicidal Self-injury and Suicidality Among Their Offspring in China
Source: JAMA Netw Open. 2021 Nov 9;4(11):e2133596. doi: 10.1001/jamanetworkopen.2021.33596 (PMC8579225; doi:10.1001/jamanetworkopen.2021.33596)
Supplement: Supplement. — eTable 1. Adolescent Social Support Scale eTable 2. Frequency and Prevalence of 8 Types of NSSI eTable 3. Odds of NSSI, Suicidal Ideation and Suicide Attempt by Migration Status in Male vs Female Participants eTable 4. Odds of NSSI, Suicidal Ideation and Suicide Attempt by Parental Migration Pattern in Male vs Female Participants eTable 5. Odds of NSSI, Suicidal Ideation and Suicide Attempt by Offspring Age When Parent Initially Migrated in Male vs Female Participants [file jamanetwopen-e2133596-s001.pdf]

Supplementary Online Content

Ma Y, Guo H, Guo S, et al. Association of the labor migration of parents with nonsuicidal self-injury and suicidality among their offspring in China. *JAMA Netw Open*. 2021;4(11):e2133596. doi:10.1001/jamanetworkopen.2021.33596

eTable 1. Adolescent Social Support Scale

eTable 2. Frequency and Prevalence of 8 Types of NSSI

eTable 3. Odds of NSSI, Suicidal Ideation and Suicide Attempt by Migration Status in Male vs Female Participants

eTable 4. Odds of NSSI, Suicidal Ideation and Suicide Attempt by Parental Migration Pattern in Male vs Female Participants

eTable 5. Odds of NSSI, Suicidal Ideation and Suicide Attempt by Offspring Age When Parent Initially Migrated in Male vs Female Participants

This supplementary material has been provided by the authors to give readers additional information about their work.

© 2021 Ma Y et al. *JAMA Network Open*.

eTable 1. Adolescent Social Support Scale

Please read each item carefully, and circle "○" on the number of options that best suits your actual situation

Items	Fully consistent	Consistent	Not sure	Inconsistent	Totally inconsistent
1. Most classmates care about me	1	2	3	4	5
2. When faced with a dilemma, I will take the initiative to ask others for help	1	2	3	4	5
3. When I have troubles, I will take the initiative to confide in my family, relatives and friends	1	2	3	4	5
4. I often get care and support from classmates and friends	1	2	3	4	5
5. When encountering difficulties, I often seek help from my family, relatives and friends	1	2	3	4	5
6. There are many close people around me who can give me support and help	1	2	3	4	5
7. When I encounter a problem, my classmates and friends will appear by my side	1	2	3	4	5
8. In times of difficulty, I can rely on my family or friends.	1	2	3	4	5
9. I often get emotional help and support from classmates and friends	1	2	3	4	5
10. I often get care and support from my family, relatives and friends.	1	2	3	4	5
11. When needed, I can get financial support from my family, relatives and friends	1	2	3	4	5
12. When in trouble, I usually take the initiative to seek help from others	1	2	3	4	5
13. When I am sick, I can always be taken care of by my family, relatives and friends.	1	2	3	4	5
14. When I have troubles, I will take the initiative to confide in my classmates and friends	1	2	3	4	5
15. When I have a problem, my family, relatives and friends will show up by my side	1	2	3	4	5
16. I often get emotional help and support from my family, relatives and friends	1	2	3	4	5
17. When encountering difficulties, I often seek help from classmates and friends	1	2	3	4	5

© 2021 Ma Y et al. *JAMA Network Open*.

eTable 2. Frequency and Prevalence of 8 Types of NSSI

Type of NSSI	Frequency of NSSI (N, %)		
	**Non-NSSI (0 time)**	**Less frequent (1-4 episodes)**	**More frequent (⩾5 episodes)**
Hitting	12786 (83.5)	2041(13.3)	485(3.2)
Head banging	14085 (92.0)	1043 (6.8)	184(1.2)
Stabbing	13635 (89.0)	1337 (8.7)	340 (2.2)
Pinching	13177 (86.1)	1687 (11.0)	448 (2.9)
Scratching	14451 (94.4)	713 (4.7)	148 (1.0)
Biting	14290 (93.3)	848 (5.5)	174 (1.1)
Burning	15121 (98.8)	161 (1.1)	30 (0.2)
Cutting	14461 (94.4)	706 (4.6)	145 (0.9)

© 2021 Ma Y et al. *JAMA Network Open*.

eTable 3. Odds of NSSI, Suicidal Ideation and Suicide Attempt by Migration Status in Male vs Female Participants

Variable	Male			Female			ROR[c]	*P* value
	**N (%)**	**OR (95%CI)[a]**	**OR (95%CI)[b]**	**N (%)**	**OR (95%CI)[a]**	**OR (95%CI)[b]**		
**Less frequent NSSI （1-4 episodes）**								
No migration	718(15.0)	1 [Reference]	1 [Reference]	765(16.8)	1 [Reference]	1 [Reference]		
Migration	558(17.9)	1.26(1.11-1.42)	1.15(1.02-1.30)	594(20.8)	1.31(1.16-1.48)	1.13(1.01-1.28)	1.02	0.20
**More frequent NSSI （⩾5 episodes）**								
No migration	536(11.2)	1 [Reference]	1 [Reference]	545(12.0)	1 [Reference]	1 [Reference]		
Migration	366(11.8)	1.11(0.96-1.28)	1.12(0.98-1.28)	336(11.8)	1.04(0.90-1.20)	0.93(0.78-1.10)	1.20	0.10
**Suicidal ideation**								
No migration	672(14.0)	1 [Reference]	1 [Reference]	738(16.2)	1 [Reference]	1 [Reference]		
Migration	435(14.0)	1.00(0.88-1.14)	0.94(0.81-1.08)	490(17.2)	1.07(0.95-1.22)	0.98(0.85-1.13)	0.96	0.17
**Suicide attempt**								
No migration	148(3.1)	1 [Reference]	1 [Reference]	168(3.7)	1 [Reference]	1 [Reference]		
Migration	110(3.5)	1.15(0.90-1.48)	1.13(0.86-1.49)	109(3.8)	1.04(081-1.33)	1.01(0.77-1.34)	1.12	0.15

a, unadjusted model.

b, adjusted for participant province, age, ethnicity, sex, single-child family, single-parent family, educational level of main caregiver, family income, parenting style and offspring social support, loneliness, psychological resilience, emotional management ability scores.

c, calculated by adjusted OR.

© 2021 Ma Y et al. *JAMA Network Open*.

eTable 4. Odds of NSSI, Suicidal Ideation and Suicide Attempt by Parental Migration Pattern in Male vs Female Participants

Variable	Male			Female			ROR[c]	*P* value
	**N (%)**	**OR (95%CI)[a]**	**OR (95%CI)[b]**	**N (%)**	**OR (95%CI)[a]**	**OR (95%CI)[b]**		
**Less frequent NSSI （1−4 episodes）**								
No migration	718(15.0)	1 [Reference]	1 [Reference]	765(16.8)	1 [Reference]	1 [Reference]		
Father migration	342(18.5)	1.32(1.14-1.52)	1.20(1.03-1.40)	369(21.3)	1.34(1.16-1.54)	1.17(1.01-1.36)	1.03	0.21
Mother migration	35(14.0)	0.94(0.65-1.36)	0.92(0.63-1.34)	36(15.7)	0.92(0.64-1.33)	0.78(0.53-1.14)	1.18	0.14
Both parent migration	181(17.8)	1.28(1.03-1.49)	1.13(0.93-1.36)	189(21.2)	1.36(1.13-1.63)	1.13(0.93-1.37)	1.00	0.25
**More frequent NSSI（≥5 episodes）**								
No migration	536(11.2)	1 [Reference]	1 [Reference]	545(12.0)	1 [Reference]	1 [Reference]		
Father migration	222(12.0)	1.15(0.97-1.36)	1.10(0.91-1.32)	194(12.2)	0.99(0.83-1.18)	0.92(0.75-1.12)	1.20	0.07
Mother migration	31(12.4)	1.11(0.75-1.65)	1.22(0.81-1.84)	28(12.2)	1.00(0.67-1.51)	0.90(0.52-1.25)	1.36	0.09
Both parent migration	113(11.1)	1.04(0.83-1.29)	1.02(0.81-1.29)	114(12.8)	1.15(0.92-1.43)	0.99(0.77-1.26)	1.03	0.22
**Suicidal ideation**								
No migration	672(14.0)	1 [Reference]	1 [Reference]	738(16.2)	1 [Reference]	1 [Reference]		
Father migration	275(14.9)	1.07(0.92-1.25)	0.99(0.84-1.17)	280(16.2)	1.00(0.86-1.16)	0.95(0.80-1.12)	1.04	0.19
Mother migration	28(11.2)	0.77(0.52-1.16)	0.83(0.54-1.26)	36(15.7)	0.96(0.67-1.38)	0.84(0.56-1.24)	0.99	0.24
Both parent migration	132(13.0)	0.92(0.75-1.12)	0.88(0.71-1.09)	174(19.5)	1.25(1.04-1.50)	1.08(0.88-1.33)	0.81	0.10
**Suicide attempt**								
No migration	148(3.1)	1 [Reference]	1 [Reference]	168(3.7)	1 [Reference]	1 [Reference]		
Father migration	68(3.7)	1.20(0.90-1.61)	1.12(0.82-1.53)	58(3.4)	0.91(0.67-1.23)	0.90(0.64-1.26)	1.24	0.10
Mother migration	11(4.4)	1.45(0.77-2.70)	1.73(0.91-3.29)	11(4.8)	1.31(0.70-2.45)	1.34(0.69-2.58)	1.29	0.15
Both parent migration	31(3.1)	0.99(0.67-1.47)	1.02(0.67-1.55)	40(4.5)	1.22(0.86-1.74)	1.12(0.76-1.65)	0.91	0.19

a, unadjusted model.

b, adjusted for participant province, age, ethnicity, sex, single-child family, single-parent family, educational level of main caregiver, family income, parenting style and offspring social support, loneliness, psychological resilience, emotional management ability scores.

c, calculated by adjusted OR

© 2021 Ma Y et al. *JAMA Network Open*.

eTable 5. Odds of NSSI, Suicidal Ideation and Suicide Attempt by Offspring Age When Parent Initially Migrated in Male vs Female Participants

Variable	Male			Female			ROR[c]	*P* value
	**N (%)**	**OR (95%CI)[a]**	**OR (95%CI)[b]**	**N (%)**	**OR (95%CI)[a]**	**OR (95%CI)[b]**		
**Less-frequent NSSI （1−4 episodes）**								
No migration	718(15.0)	1 [Reference]	1 [Reference]	765(16.8)	1 [Reference]	1 [Reference]		
Preschool age (≤ 6y)	248(18.6)	1.36(1.16-1.60)	1.19(1.01-1.41)	291(21.8)	1.42(1.22-1.66)	1.16(1.01-1.37)	1.03	0.21
School age (6y~10y)	224(17.7)	1.20(1.02-1.42)	1.10(0.92-1.30)	216(20.4)	1.26(1.06-1.49)	1.12(0.93-1.34)	0.98	0.22
Adolescence (>10y)	86(17.0)	1.16(0.90-1.49)	1.16(0.90-1.49)	87(18.9)	1.14(0.89-1.46)	1.08(0.83-1.40)	1.07	0.18
**More-frequent NSSI （≥5 episodes）**								
No migration	536(11.2)	1 [Reference]	1 [Reference]	545(12.0)	1 [Reference]	1 [Reference]		
Preschool age (≤ 6y)	186(14.0)	1.37(1.14-1.64)	1.27(1.04-1.55)	171(12.8)	1.17(0.97-1.41)	0.96(0.77-1.19)	1.32	0.02
School age (6y~10y)	125(9.9)	0.90(0.72-1.11)	0.90(0.72-1.13)	115(10.8)	0.94(0.76-1.16)	0.88(0.69-1.11)	1.02	0.23
Adolescence (>10y)	55(10.8)	0.99(0.74-1.34)	1.05(0.77-1.43)	50(10.8)	0.92(0.67-1.25)	0.97(0.70-1.35)	1.08	0.19
**Suicidal ideation**								
No migration	672(14.0)	1 [Reference]	1 [Reference]	738(16.2)	1 [Reference]	1 [Reference]		
Preschool age (≤ 6y)	215(16.1)	1.18(1.00-1.40)	1.03(0.86-1.24)	263(19.7)	1.27(1.09-1.49)	1.09(0.91-1.30)	0.94	0.17
School age (6y~10y)	163(12.8)	0.90(0.75-1.09)	0.86(0.71-1.05)	166(15.6)	0.96(0.80-1.15)	0.93(0.76-1.13)	0.92	0.15
Adolescence (>10y)	57(11.2)	0.78(0.58-1.04)	0.80(0.59-1.08)	61(13.2)	0.79(0.60-1.04)	0.83(0.62-1.12)	0.96	0.22
**Suicide attempt**								
No migration	148(3.1)	1 [Reference]	1 [Reference]	168(3.7)	1 [Reference]	1 [Reference]		
Preschool age (≤ 6y)	55(4.1)	1.35(0.99-1.86)	1.22(0.87-1.72)	60(4.5)	1.23(0.91-1.66)	1.07(0.76-1.51)	1.14	0.15
School age (6y~10y)	37(2.9)	0.94(0.65-1.36)	0.96(0.66-1.41)	30(2.8)	0.76(0.51-1.13)	0.79(0.52-1.19)	1.22	0.13
Adolescence (>10y)	18(3.6)	1.16(0.70-1.90)	1.28(0.77-2.15)	19(4.1)	1.12(0.69-1.82)	1.36(0.82-2.24)	0.94	0.22

a, unadjusted model.

b, adjusted for participant province, age, ethnicity, sex, single-child family, single-parent family, educational level of main caregiver, family income, parenting style and offspring social support, loneliness, psychological resilience, emotional management ability scores.

c, calculated by adjusted OR.

© 2021 Ma Y et al. *JAMA Network Open*.
